# First case of Lucio's phenomenon in a lepromatous leprosy patient following COVID-19 viral vector vaccine

**DOI:** 10.17179/excli2023-6661

**Published:** 2023-10-30

**Authors:** Roberto Fernandes Soares-Neto, Geyse Maria Lima da Piedade, Priscila Soares Pereira, Rebeca Yasmin Ribeiro Vieira, Jerocilio Maciel de Oliveira-Júnior, Thalyta Porto Fraga, Martha Débora Lira Tenório, Pedro Dantas Oliveira, Paulo Ricardo Martins-Filho

**Affiliations:** 1 Residency Program in Dermatology, University Hospital, Federal University of Sergipe, Brazil; 2 Residency Program in Infectious Diseases, University Hospital, Federal University of Sergipe, Brazil; 3 Graduate Program in Health Sciences, Federal University of Sergipe, Brazil; 4 Investigative Pathology Laboratory, Federal University of Sergipe, Brazil; 5 Laboratory of Pathological Anatomy, University Hospital, Federal University of Sergipe, Brazil; 6 Department of Medicine, Federal University of Sergipe, Brazil

## ⁯⁯⁯

Since the introduction of COVID-19 vaccines, several cases of thrombotic, allergic, and immunological exacerbations have been documented (Rerknimitr et al., 2022[3]). However, literature scarcely addresses post-vaccination repercussions in leprosy patients. Herein, we discuss a 45-year-old female patient with lepromatous leprosy (LL) who manifested erythematous lesions seven days post her second dose of the Oxford-AstraZeneca non-replicating viral-vectored vaccine (ChAdOx1 nCoV-19), which evolved into necrotic ulcers on her limbs, abdomen, and back (Supplementary Figure 1A-C). At the vaccination time, she was three months into her multibacillary multidrug therapy (MB-MDT), hailing from Sergipe, Northeast Brazil - an area identified as a leprosy endemic region in the country.

Upon hospital admission, diagnostic evaluations showed anemia, leukocytosis, elevated C-reactive protein and erythrocyte sedimentation, negative tests for SARS-CoV-2, hepatitis B, C, HIV, and syphilis. An ulcer biopsy depicted a moderate lymphohistiocytic inflammatory infiltration in the superficial and deep dermis, as well as around arteries, skin appendages, and nerves. The Ziehl-Neelsen stain accentuated fragmented and intact acid-fast bacilli (AFB), remarkably in endothelial cells and vessel walls. There was no neutrophilia or panniculitis in the sample (Supplementary Figure 1D-I). This histological pattern confirmed the diagnosis of Lucio's Phenomenon (LP). Following a therapeutic regimen including broad-spectrum antibiotic therapy, thalidomide 300 mg/day, prednisone 1-2 mg/kg/day, and continued MB-MDT, she evidenced notable lesion regression within two weeks.

LP, a necrotizing vasculopathy associated with coagulation abnormalities, was first detailed in diffuse leprosy patients by Lucio and Latapi. Clinically, this reaction typifies as ulcerating erythematous/cyanotic lesions, with or without systemic symptoms. Its etiology, while unclear, postulates a thrombotic state arising from bacillary endothelial colonization. The histopathological diagnosis is based especially on the presence of (1) vascular thrombosis in cutaneous arteries, potentially leading to necrosis and ulceration; and (2) intact and fragmented AFB penetration into endothelial cells. In the early stages of LP, lymphohistiocytic inflammatory infiltration is absent; however, it may manifest in the dermis of mature lesions as a post-necrotic inflammatory reaction (Benard et al., 2009[1]). The primary differential diagnosis for LP is Erythema Nodosum Leprosum necroticans, defined by its distinct neutrophilic dermohypodermic inflammatory infiltration within small to medium-sized arteries and the presence of fragmented AFB in endothelial cells and histiocytes.

Leprosy reactions are known to be triggered by factors such as MDT initiation, pregnancy, infections, and stress. Vaccinations, specifically against smallpox, BCG, *Mycobacterium indicus pranii*, influenza, and hepatitis B, have also been identified as potential precipitants. Recently, case reports have described the occurrence of Erythema Nodosum Leprosum following ChAdOx1 nCoV‐19 vaccine (Bhandari et al., 2022[2]); however, to date, no cases of LP have been reported post-administration of this vaccine technology. ChAdOx1 nCoV-19, a monovalent vaccine, utilizes a replication-deficient recombinant chimpanzee adenovirus expressing SARS-CoV-2's S-glycoprotein (S-gp). S-gp triggers the production of neutralizing antibodies and a Th1 immune response, resulting in elevated levels of TNF-alpha, INF-gamma, and IL-8 (Sahin et al., 2020[4]). The presence of immunocomplexes containing S-gp in the endothelium has been suggested to induce endothelitis in cases of leukocytoclastic vasculitis instigate by such vaccines (Sandhu et al., 2021[5]). Our case presumably mirrors this pathophysiological trajectory, where BAAR-invaded endothelium results in cutaneous infarction and vasculonecrotic lesions. 

This report presents the first LP case following ChAdOx1 nCoV-19 vaccination. However, potential coincidental links between LP and this vaccine should be approached cautiously. Additionally, adverse reactions shouldn't overshadow the proven safety, efficacy, and benefits of COVID-19 vaccines. This observation reinforces the imperative for vigilant monitoring of post-vaccination adverse events, especially in leprosy-prevalent regions.

## Declaration

### Conflict of interest

There is no conflict of interest to declare.

### Funding

There is no funding source.

## Supplementary Material

Supplementary information

## References

[1] Benard G, Sakai-Valente NY, Bianconcini Trindade MA (2009). Concomitant Lucio phenomenon and erythema nodosum in a leprosy patient: clues for their distinct pathogeneses. Am J Dermatopathol.

[2] Bhandari A, Shilpa, Gupta S, Dogra S, Narang T (2022). Reactions in leprosy patients triggered by COVID‐19 vaccination – a cross‐sectional study from a tertiary care centre in India. J Eur Acad Dermatology Venereol.

[3] Rerknimitr P, Puaratanaarunkon T, Wongtada C, Wittayabusarakam N, Krithin S, Paitoonpong L (2022). Cutaneous adverse reactions from 35,229 doses of Sinovac and AstraZeneca COVID‐19 vaccination: a prospective cohort study in healthcare workers. J Eur Acad Dermatology Venereol.

[4] Sahin U, Muik A, Derhovanessian E, Vogler I, Kranz LM, Vormehr M (2020). COVID-19 vaccine BNT162b1 elicits human antibody and TH1 T cell responses. Nature.

[5] Sandhu S, Bhatnagar A, Kumar H, Dixit PK, Paliwal G, Suhag DK (2021). Leukocytoclastic vasculitis as a cutaneous manifestation of ChAdOx1 nCoV‐19 corona virus vaccine (recombinant). Dermatol Ther.

