# Supplementary material for: First case of Lucio's phenomenon in a lepromatous leprosy patient following COVID-19 viral vector vaccine
Source: EXCLI J. 2023 Oct 30;22:1132–4. doi: 10.17179/excli2023-6661 (PMC10711195; doi:10.17179/excli2023-6661)
Supplement: Supplementary information [file EXCLI-22-1132-s-001.pdf]

EXCLI Journal 2023;22:1132-1134 – ISSN 1611-2156
Received: October 03, 2023, accepted: October 16, 2023, published: October 30, 2023

Supplementary information to:

Letter to the editor:

FIRST CASE OF LUCIO'S PHENOMENON IN A LEPROMATOUS LEPROSY PATIENT FOLLOWING COVID-19 VIRAL VECTOR VACCINE

Roberto Fernandes Soares-Neto[1], Geyse Maria Lima da Piedade[1], Priscila Soares Pereira[1], Rebeca Yasmin Ribeiro Vieira[2], Jerocilio Maciel de Oliveira-Júnior[3,4], Thalyta Porto Fraga[5], Martha Débora Lira Tenório[1,3,4], Pedro Dantas Oliveira[6], Paulo Ricardo Martins-Filho[3,4*]

1 Residency Program in Dermatology, University Hospital, Federal University of Sergipe, Brazil
2 Residency Program in Infectious Diseases, University Hospital, Federal University of Sergipe, Brazil
3 Graduate Program in Health Sciences, Federal University of Sergipe, Brazil
4 Investigative Pathology Laboratory, Federal University of Sergipe, Brazil
5 Laboratory of Pathological Anatomy, University Hospital, Federal University of Sergipe, Brazil
6 Department of Medicine, Federal University of Sergipe, Brazil

* **Corresponding author:** Prof. Paulo Ricardo Martins-Filho, Universidade Federal de Sergipe, Hospital Universitário, Laboratório de Patologia Investigativa. Rua Cláudio Batista, s/n. Sanatório. Aracaju, Sergipe, Brasil. CEP: 49060-100.
E-mail: prmartinsfh@gmail.com

https://dx.doi.org/10.17179/excli2023-6661

This is an Open Access article distributed under the terms of the Creative Commons Attribution License (http://creativecommons.org/licenses/by/4.0/).

EXCLI Journal 2023;22:1132-1134 – ISSN 1611-2156
Received: October 03, 2023, accepted: October 16, 2023, published: October 30, 2023

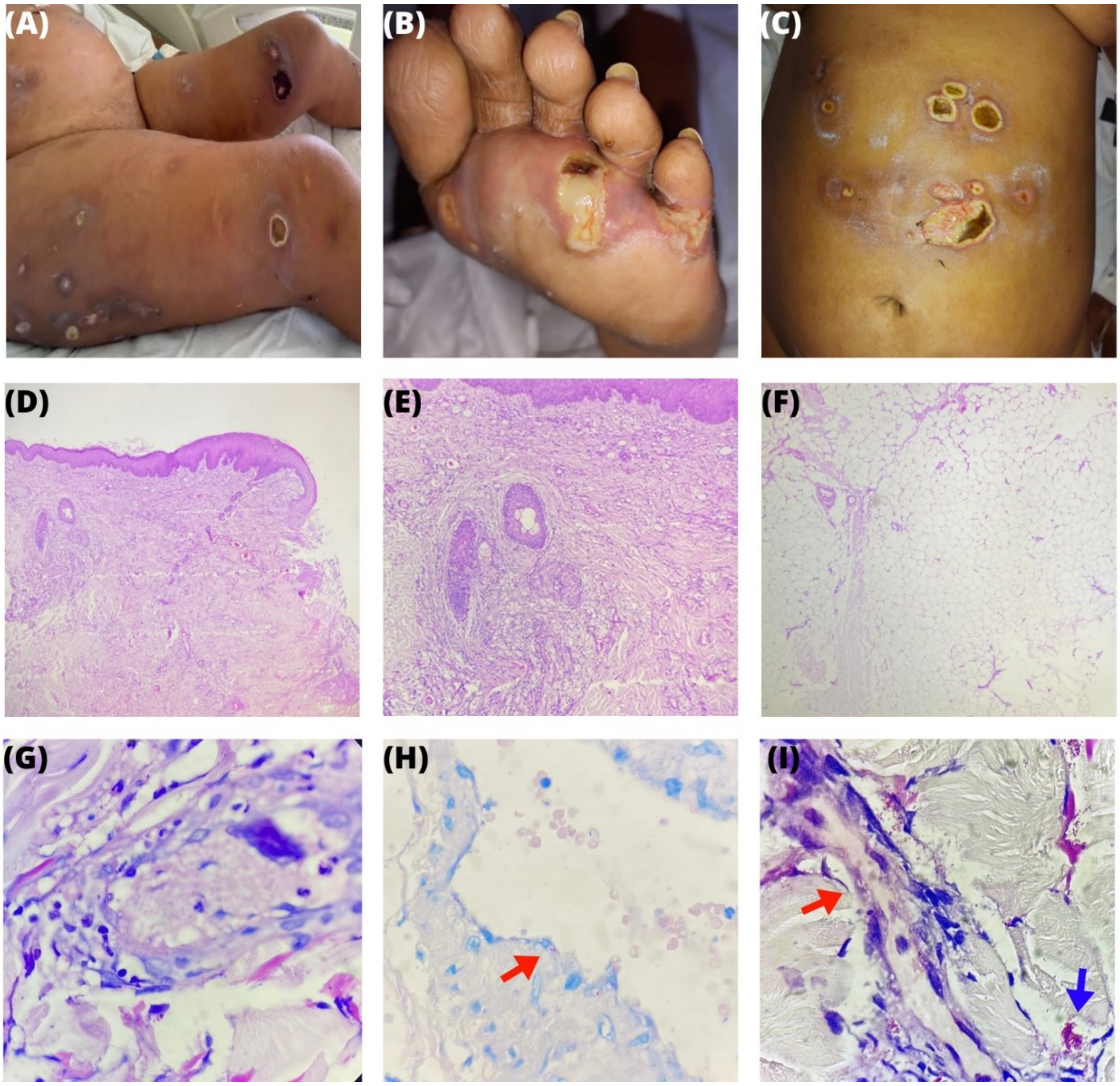

Supplementary Figure 1: (**A-C**) Necrotic ulcers with violaceous erythematous borders on the abdomen and lower limbs; (**D**) Peripheral ulceration site showcasing a moderate dermal lymphohistiocytic inflammatory infiltrate (H-E stain, ×100); (**E**) Lymphohistiocytic inflammatory presence around pilosebaceous follicles (H-E stain, ×100); (**F**) Intact hypodermis with sporadic lymphohistiocytic inflammatory foci surrounding blood vessels (H-E stain, ×100); (**G**) Vessel depicted with mixed inflammatory infiltration within its wall and noticeable moderate edema; an adherent thrombus is visible within the lumen (H-E stain, ×400); (**H-I**) Ziehl-Neelsen staining reveals abundant AFBs, a mix of fragmented and intact. Red arrows pinpoint an AFB within the vessel endothelium, while a blue arrow indicates globias (Ziehl-Neelsen stain, ×400).
